# Delayed regression of laser‐induced choroidal neovascularization in TNFα‐null mice

**DOI:** 10.1111/jcmm.17562

**Published:** 2022-09-20

**Authors:** Hiroki Iwanishi, Osamu Yamanaka, Takayoshi Sumioka, Shingo Yasuda, Masayasu Miyajima, Shizuya Saika

**Affiliations:** ^1^ Department of Ophthalmology Wakayama Medical University Wakayama Japan

**Keywords:** apoptosis, choroidal neovascularization, neutrophil, tumour necrosis factor α

## Abstract

We investigated the effects of lacking TNFα on the development and regression of Argon‐laser‐induced choroidal neovascularization (CNV) in mice. We lasered ocular fundus for induction of CNV in both wild‐type (WT) and TNFα‐null (KO) mice. Fluorescence angiography was performed to examine the size of CNV lesions. Gene expression pattern of wound healing‐related components was examined. The effects of exogenous TNFα on apoptosis of human retinal microvascular endothelial cells (HRMECs) and on the tube‐like structure of the cells were investigated in vitro. The results showed that Argon‐laser irradiation‐induced CNV was significantly larger in KO mice than WT mice on Day 21, but not at other timepoints. Lacking TNFα increased neutrophil population in the lesion. The distribution of cleaved caspase3‐labelled apoptotic cells was more frequently observed in the laser‐irradiated tissue in a WT mouse as compared with a KO mouse. Exogenous TNFα induced apoptosis of HRMECs and accelerated regression of tube‐like structure of HRMECs in cell culture. Taken together, TNFα gene knockout delays the regression of laser‐induced CNV in mice. The mechanism underlying the phenotype might include the augmentation of neutrophil population in the treated tissue and attenuation of vascular endothelial cell apoptosis.

## 1 INTRODUCTION

Age‐related macular degeneration (AMD) is one of the major diseases with potential severe impairment of vision.^1^, ^2^ It includes two types; macular degeneration with macular neovascularization and atrophy of the macular retinal pigment epithelium and choroid without CNV. Macular neovascularization that grows from choroidal tissue is usually associated with severe exudate and haemorrhage, both of which cause severe vision disturbance.^3^ Although intravitreal administration of anti‐vascular endothelial growth factor (VEGF) antibody exerts a powerful therapeutic efficacy, the presence of macular neovascularization refractory to the therapy or growth of subretinal fibrosis in the macular region are to be investigated. Understanding the network system of growth factors/cytokines that governs the development of macular neovascularization and related tissue fibrosis is essential to overcome the remaining problems.

In vivo research that targets AMD, macular neovascularization is usually modelled by choroidal neovascularization (CNV) induced by Argon laser irradiation in mice. Inflammation with neutrophils and macrophages plays a crucial role in the development of CNV.^4^ Immune complex deposition and complement activation under the condition of tissue inflammation were proposed as important mediators of macular neovascularization or CNV growth.^5^, ^6^, ^7^ We reported that the loss of Smad3, the major transforming growth factor β (TGFβ) signalling transmitter, suppressed the growth of argon laser‐induced CNV in association with inhibition of local tissue inflammation in mice.^8^

Infiltration of macrophages was detected in a surgically removed CNV tissue, and the macrophages were labelled for not only VEGF but also one of the major pro‐inflammatory cytokines, tumour necrosis factor α (TNFα), suggesting that TNFα also contributes to the pathophysiology of CNV in AMD.^9^ Blocking TNFα activity by administrating a neutralizing antibody exhibits therapeutic potential in inflammatory fibrotic diseases in various organs, for example, lung or joints in mice.^10^, ^11^ These reports strongly suggest that partial attenuation of TNFα signalling slowdowns the process of inflammatory fibrosis in pathological conditions, for example, rheumatoid arthritis.^12^, ^13^ Local administration of anti‐TNFα antibody inhibits the development of laser‐induced CNV in mice.^14^, ^15^, ^16^ In human patients, Shi et al. reported that a TNFα‐trapping peptide, etanercept, or a TNFα antibody reduced laser‐induced CNV.^17^ Systemic or intracameral infliximab, an anti‐TNFα monoclonal antibody, was reported to be effective against age‐related AMD^15^, ^16^ but did not exhibit an additional effect in patients treated with anti‐VEGF antibody.^18^ TNFα exerts a variety of biological activities by signalling via its two receptors, TNFR‐1 and TNFR‐2. Jasielska et al. reported that lacking TNFR‐1 did not suppress, or even promotes, the development of laser‐induced CNV and that deletion of TNFR‐2 gene suppresses its growth.^19^ In other tissue of the body, blocking TNFα is a powerful therapeutic option in the suppression of immune‐related inflammatory disorders.

On the contrary, complete inhibition of TNFα signalling by ligand gene knockout reportedly paradoxically enhances the inflammatory process in a variety of disease models depending on tissues in animals.^20^, ^21^ It was also reported that TNFα accelerates the resolution of tissue fibrosis in a mouse lung.^22^ TNFα in lymphocytes suppress tissue fibrosis in a mouse kidney.^23^ We previously showed that the loss of TNFα promotes excess inflammation and resultant tissue scarring in corneal stroma during healing following an alkali burn in mice.^24^ These reports suggest that partial neutralization of TNFα ligand does not recapitulate the phenotype by total loss of TNFα by gene knockout.

In the current study, we investigated the effects of the loss of TNFα on the development and regression of argon‐laser‐induced CNV in mice in order to better understand the roles of this growth factor in CNV pathobiology. These reports above mentioned promoted us to hypothesize that complete deletion of TNFα could lead to excess or could not affect the growth of CNV following laser irradiation in mice. The main outcome indicated that lacking TNFα gene does not suppress the growth of CNV but delays the regression of the CNV tissue in mice.

## 2 MATERIALS AND METHODS

Experiments were approved by the DNA Recombination Experiment Committee and the Animal Care and Use Committee of Wakayama Medical University. They were conducted in accordance with the ARVO Statement for the Use of Animals in Ophthalmic and Vision Research.

### 2.1 Mice

Male adult (6–8‐week‐old) C57BL/6 (WT) mice and TNFα‐null (KO) mice of the same background were used. *Tnf*α^−/−^ mouse in C57BL/6 genetic background (KO mouse) was a generous gift from H. Tsutsui (Hyogo Medical University).^25^

### 2.2 Experimental argon‐laser‐induced CNV model in mice

Argon laser irradiation‐induced CNV is the established mouse model for human AMD, although a mouse does not have macula. We irradiated ocular fundus for induction of CNV by using Argon laser in both male WT (*n* = 25) and male KO (*n* = 25) mice according to the procedure, we previously reported with a minor modification.^8^ Under general anaesthesia induced by the inhalation of 2% isoflurane (Mylan), we irradiated ocular fundus in each quadrant of a mouse (Power, 200 mW; spot size, 80 μm; duration, 0,05 s.; NIDEK). The procedure of irradiation in both eyes was approved by the Animal Care and Use Committee of Wakayama Medical University. A mouse does not have macula, and thus, it is possible to produce four laser irradiation spots in both eyes. Seven, 14, 21, 28 and 42 days after the laser irradiation (5 mice at each timepoint), the CNV was visualized with fluorescein isothiocyanate (FITC)‐dextran angiography; FITC‐dextran (1 ml/mouse, 50 mg/ml; 2 × 10^6^ MW; Sigma, 10% dextran, FITC: PBS = 1:1 weight ratio) was introduced into the cardiac cavity according to our previous publication.^8^ The mice were killed, and the eyes were enucleated.^8^ CNV in a flat‐mounted choroidal specimen was then observed under Apotome2 fluorescent microscope (Zeiss, Germany) after removing the cornea, lens and sclera.^8^ The hyper‐fluorescent tissues of CNV lesions (referred to as the size of CNV) were measured, and the total area of CNV lesions per eye was determined as their sum.^8^ A constant threshold in pixels (corresponding to threshold fluorescence) was used to quantify neovascularization. The total hyperfluorescent area of CNV was measured by using the software of WinROOF (Mitani, Japan).^8^ In brief, FITC‐visualized CNV was shown in RGB colour in the software of WinROOF and the area of FITC staining (not the whole area, but just the vessels) was determined. A two‐sample Student's *t*‐test with unequal variance was used for statistical analyses of the quantitative CNV flat‐mount data. Experiments were performed according to the previous publications by the authors and others.^8^, ^24^

### 2.3 Gene expression analysis

Uninjured mice and the mice on Days 1, 3, 5, 7 and 9 days after the laser irradiation were sacrificed and the eye was enucleated. Ten WT and 10 KO mice (20 eyes at each timepoint in each genotype of mice) were used at each day‐point. Chorio‐sclera complex tissue was isolated by removal of the cornea, lens and retina because choroidal tissue and sclera tightly adhered to each other. We consider mRNA derived from sclera does not significantly affect the data because cellular components in the sclera was minimal. The samples were processed for RNA extraction by using a Sigma kit, GenElute™ Mammalian Total RNA Miniprep Kit (RTN70‐1Kit, Lot # SLBS6196). Four samples were merged into one tube. Totally, sixty WT and 60 KO mice were used to obtain their RNA samples. Real‐time RT‐PCR (TaqMan) was performed in order to semi‐quantify the mRNA expression level of F4/80, VEGF‐A, interleukin‐6 (IL‐6), TGFβ1, macrophage‐chemoattractant protein‐1 (MCP‐1), myeloperoxidase (MPO, a neutrophil marker), matrix metalloproteinase‐2 (MMP2) and MMP9 with the expression level of internal control, glyceraldehyde‐3‐phosphate dehydrogenase (GAPDH). TaqMan primers for mRNAs of F4/80 (Mm00802529_ml), VEGF‐A (Mm01281447_ml), IL‐6 (Mm01210732_ml), TGFβ1 (Mm03024053_ml), MCP‐1 (Mm9999056_ml), MPO (Mm01298422_gl), MMP‐2 (Mm00439498_ml) and MMP‐9 (Mm0044299_ml) were purchased from Applied Biosystems (Drive Foster City). Data were analysed delta/delta C1 method (Applied Biochemistry Inc.) and Mann–Whitney *U*‐test with a significant level of *p* < 0.05.

### 2.4 Histology and immunohistochemistry

Real‐time‐PCR showed the marked difference of expression pattern of MPO mRNA on Day 3, and we performed immunohistochemistry for neutrophils in flat‐mounted specimens of chorioretinal tissues (*n* = 5 in each genotype of mice) in order to quantify the numbers of the cells in a flat‐mounted tissue specimen on Day 4. The number of the laser spots at each eye was 4.

All the samples were fixed in 4% paraformaldehyde for 10 min and processed for immunohistochemistry with a rat monoclonal anti‐LY6B.2 antibody. Alloantigen Antibody [a marker for a neutrophil leucocyte, 1:100 dilution in phosphate‐buffered saline (PBS), Bio‐Rad Laboratories] as previously reported.^8^

It was reported that apoptotic cell death was observed following the peak of the growth of laser‐induced CNV in mice.^2^ Four laser irradiation spots were produced in two eyes of each genotype of mouse (totally 8spots in each genotype). Flat‐mounted chorioretinal tissues of each genotype of mice on Days 13, 15 and 17 post‐laser irradiation were processed for double‐immunohistochemistry by using an anti‐CD31 antibody (PECAM 1:100; sc‐18,916; Santa Cruz Biotechnology) and cleaved caspase3 (Asp175) [5A1E, 1:100 dilution in PBS, Cell Signalling, USA]. Visualization was performed with FITC‐ or Alexa Fluor 647 (pink, Invitrogen, A35173)‐conjugated secondary antibody.

### 2.5 mRNA Expression of TGFβ1 or VEGF‐A in macrophages of neutrophils

Mouse macrophages were obtained from the peritoneal cavity using a glycogen stimulation method, as previously reported by us.^24^ In brief, 5% sterilized oyster glycogen (Sigma‐Aldrich) in phosphate‐buffered saline (PBS, 1 ml) was injected into the peritoneal space of either a WT (*n* = 5) or KO (*n* = 5) mouse aged 6–8 weeks. After 4 days, the peritoneal cavity was irrigated with Eagle's medium to harvest macrophages. Approximately 90% of the cells obtained by this method were positive for F4/80 as previously reported by us.^24^ The cells were allowed to adhere to 60 mm culture dishes for 6 h, and then, nonadherent cells were washed out with PBS. RNA extracted from the adherent cells (macrophages) were processed TaqMan real‐time RT‐PCR for mRNA of TGFβ1 or VEGF‐A. Three specimens were prepared for each condition. Five dishes were prepared for each culture condition.

Mouse neutrophils were harvested as follows.^24^ 5% (w/v) sterilized casein (Sigma‐Aldrich) in phosphate‐buffered saline (PBS, 2 ml) was injected into the peritoneal space of either a WT (*n* = 5) or KO (*n* = 5) mouse aged 6–8 weeks. After 3 h, the peritoneal cavity was irrigated with Eagle's medium to harvest neutrophils. Previous publications reported^26^ that approximately over 90% of the cells obtained were neutrophils, which was also confirmed Giemsa staining (data not shown). The cells were allowed to adhere to 60 mm culture dishes for 6 h, and then nonadherent cells were washed out with PBS. RNA extracted from the adherent cells (neutrophils) were processed TaqMan real‐time RT‐PCR for mRNA of VEGF‐A. Five specimens were prepared for each condition. Data at each time point were statistically analysed using the *Mann–Whitney U test*.

### 2.6 Culture of a human retinal microvascular endothelial cell (HRMEC)

Frozen HRMECs (Commercially available primary cultured cells from healthy donner isolated by elutriation of dispase dissociated normal human retina., Distributor: DS Pharma Japan, Manufacturer: Cell Systems, Cat# CS‐ACBRI 181, Lot# 181.01.03.01.02) were recovered on the specific 75 cm^2^ culture bottle (Corning, Cat# 3276) in the specific serum‐containing CS‐CC basic medium (Distributor: DS Pharma Japan, Manufacturer: Cell Systems, Cat# CS‐4Z0‐500R) supplemented with a defined cell boost (Distributor: DS Pharma Japan, Manufacturer: Cell Systems, Cat# CS‐4CB‐500R) and antibiotics (100 U/ml of penicillin and 100 μg/ml of streptomycin) in a CO_2_ incubator. The cells of 80% confluency were used for experiments. The culture dishes were treated with Attachment factor, 4ZO‐210 (Distributor: DS Pharma Japan, Manufacturer: Cell Systems).

### 2.7 Apoptosis of HRMECs in the presence of TNFα

HRMECs were plated in wells of a regular 96‐well plate (Corning, Cat. No. NCO3904) at a concentration of 2.0 × 10^4^ cells/well in the medium mentioned above. After incubation for 24 h, cells were treated with 0, 1, 5, 10 ng/ml TNFα (0, 1, 5 or 10 ng/ml, R&D. Cat#. 210‐TA‐020/CF, 4 wells for each condition) in a serum‐free specific medium (CS‐C medium). After 6 or 21 h treatment, cells were stained for annexin V and PI using Dead Cell Apoptosis Kit (Thermo‐Fisher Scientific, Cat. No. V13241) or Hoechst (Thermo‐Fisher Scientific, Cat. No. V13241). The kit contains recombinant annexin V conjugated to fluorescein (FITC annexin V), as well as a solution of the red‐fluorescent propidium iodide (PI) nucleic acid binding dye for apoptosis detection. PI is impermeant to live cells and apoptotic cells, but stains dead cells with red fluorescence, binding tightly to the nucleic acids in the cell. After staining a cell population with FITC annexin V and PI, apoptotic cells show green fluorescence, dead cells show red and green fluorescence and live cells show little or no fluorescence. A previous report suggested caspase 3/8 was activated at 6 h upon TNFα exposure.^14^ Thus, we defined annexin V‐negative and PI‐negative cells as live cells, annexin V‐positive and PI‐negative cells as early apoptotic cells, annexin V‐positive and PI‐positive cells as cells at the later stage of apoptosis. The kit is originally for flow cytometry, it was also applied to the direct observation of apoptotic cells. In the current study, we directly observed immunolabelled cells under fluorescence microscopy (Keyence, BZ‐X700) and software‐assisted analysis (Operetta CLS, Perkin Elmer) were performed to count labelled cells to examine the effects of supplementation of TNFα on cell apoptosis. Statistical analysis was performed using Student's t‐test with a significant level of *p* < 0.05.

### 2.8 Effects of exogenous TNFα on the tube‐like structure of HRMECs in vitro

HRMECs (1 × 10^6^ cells/25 cm^2^) were cultured in a T75 culture bottle for 24 h. At 80% confluency, the cells were washed in PBS and then further incubated for 30 min in a serum‐free medium containing 2 μM Calcein AM. The cells were then trypsinized and were collected by a centrifugal separator (1000 × *g*, 5 min). Cultrex® Reduced Growth Factor Basement Membrane Extract (10 μl, Trevigen®) was placed in wells of a 96‐well plate and allowed to set for 30 min in a CO_2_ incubator. Cells (2 × 10^4^ cells in 70 μl/well) were seeded and incubated for 10 h for tube‐like structure formation. Then, TNFα at the final concentrations of 1, 5 or 10 ng/ml was administered, and the morphology of the tube‐like structure was observed until 10 h under time‐lapse microscopy. Four wells were prepared for each culture condition. The total length of the tube‐like structure was measured as pixels in each condition at time points by an observation under microscopy (Keyence, BZ‐X700) and statistically analysed by using the Mann–Whitney *U*‐test.

## 3 RESULTS

### 3.1 Experimental argon‐laser‐induced CNV model

**FIGURE 1:**
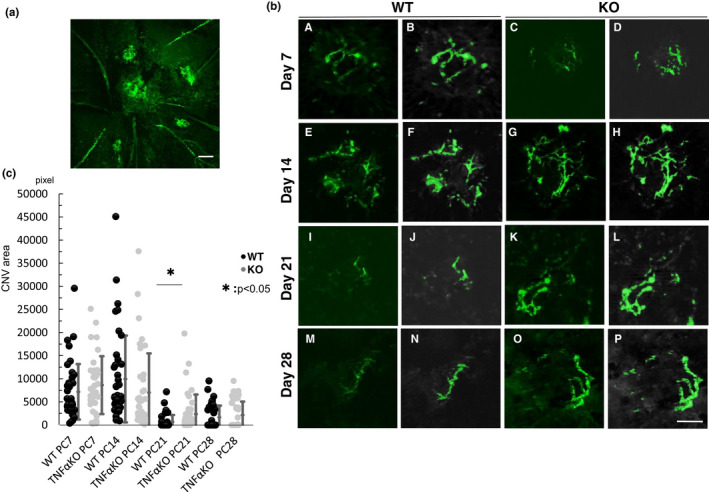
Loss of tumour necrosis factor α (TNFα) delayed the regression of the laser‐induced choroidal neovascularization (CNV). (a). Typical angiography with fluorescein isothiocyanate (FITC)‐labelled dextran in choroidal flat‐mounted specimens show the presence of 4 spots of CNV in wild‐type (WT) on Day 14. Bar, 200 μm. (b). Angiography with FITC‐labelled dextran in choroidal flat‐mounted specimens show the presence of CNV in both wild‐type (WT) and TNFα‐null (KO) tissues (A–F) at Days 7, 14, 21 and 28. The size of FITC‐labelled CNV is smaller in a WT choroid (e) as compared with a KO tissue at Day 21. Bar, 50 μm. Frames A, E, I, M were representative images obtained by fluorescence microscopy in WT eyes and those of C, G, K and O show images in KO tissues. Frame B, F, J, N shows CNV area in WT eyes analysing by computer software and those of D, H, L and P shows those in KO tissues, respectively. (c). Computer software‐assisted analysis shows that the size of CNV is significantly larger in KO mice than that in WT animals at day 21. Y axis indicates total pixels measured. Bar, s.e.m.; *p < 0.05 by the Student's *t*‐test

CNV in a flat‐mounted choroidal specimen was then observed under Apotome2 fluorescent microscope (Zeiss) after removal of the cornea, lens and sclera. As previously reported, laser irradiation successfully induced CNV (Figure 1a).^8^ The hyper‐fluorescent tissues (not the whole area, but just the vessels) of CNV lesions (referred to as the size of CNV) were measured, and the total area of CNV lesions per eye was determined as their sum as described above.^8^ The size of induced CNV increased until Day 14 and then began to regress in a WT mouse. The CNV disappeared on Days 28–56 (data not shown). Similarly, CNV was well induced in KO mice with a peak of the CNV size at day 14 and then became smaller on Day 21. Statistical analysis showed that size of laser‐induced CNV formation was significantly larger in KO mice on Day 21, but not other timepoints (Days 7, 14 and 28), than in WT mice (*p* < 0.05) (Figure 1b,c). On Day 56, CNV was no longer observed in either genotype of mice (data not shown).

### 3.2 Inflammatory cell infiltration

**FIGURE 2:**
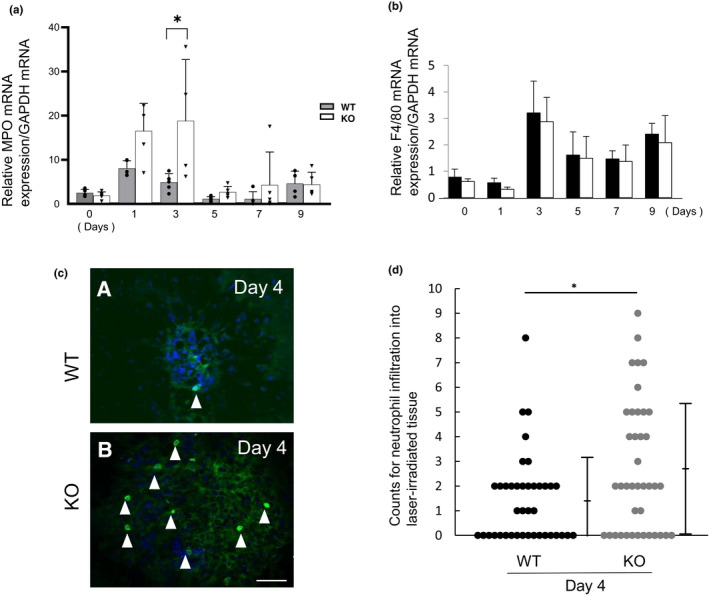
Loss of tumour necrosis factor α (TNFα) augments the population of neutrophils, but not macrophages, in the chorioretinal tissue following laser‐irradiation. (a). mRNA expression evaluation showed expression of myeloperoxidase (MPO, a neutrophil marker) was more marked in the choroidal tissue on Day 3 following laser‐irradiation in TNFα‐null (KO) tissues as compared with in wild‐type (WT) mice. (b). Expression of F4/80 mRNA (macrophage marker) was not altered at each timepoint post‐laser irradiation in a KO mouse as compared with a WT mouse. Bars, s.e.m.; *p < 0.05, by the Mann–Whitney *U*‐test. (c). Immuno‐detection of LB6B.2 antigen (a neutrophil marker) detects immunolabelled neutrophils more frequently in a KO tissue (B) as compared with a WT mouse in a laser‐irradiated area of the choroidal neovascularization on Day 4. Nuclei were labelled with DAPI. Bar, 50 μm. (d). The numbers of neutrophils in laser‐irradiated area of the choroidal tissues in flat‐mounted specimens at day 4. Neutrophil infiltration number is significantly higher in a KO tissue as compared with a WT eye. *p < 0.05 by the one‐way anova

Gene expression analysis by RT‐PCR showed that laser irradiation upregulated mRNA expression of a neutrophil marker (MPO) (Figure 2a) followed by a gradual decrease in tissue.

Expression of F4/80 macrophage marker was not affected by the loss of TNFα (Figure 2b).

Immunohistochemistry in a flat‐mounted chorioretinal specimen showed more LY6B.2‐labelled neutrophils in the KO tissue as compared with a WT tissue on Day 4 (Figure 2c). Frame d in Figure 2 shows the numbers of neutrophils in laser‐irradiated area of the choroidal tissues in flat‐mounted specimens were significantly more in the KO tissue as compared with a WT tissue at day 4 (*p* < 0.05). The number of eyes used was 5 in each genotype of mice and 4 laser spots were placed in each eye.

### 3.3 Expression of wound healing‐related components

**FIGURE 3:**
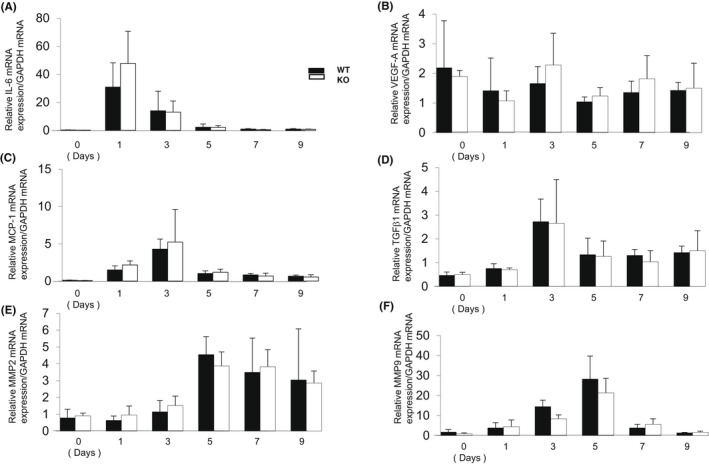
Loss of tumour necrosis factor α (TNFα) did not altered mRNA expression of inflammatory cytokines, matrix metalloproteinase (MMP)‐2 and MMP‐9 during the development of laser‐induced choroidal neovascularization (CNV). There was no significant difference of mRNA expression level of interleukin‐6 (IL‐6, A), vascular endothelial growth factor A (VEGF‐A, B), monocyte chemotactic protein‐1 (MCP‐1, C), transforming growth factor β1 (TGFβ1, D), matrix metalloproteinase‐2 (MMP‐2, E) and MMP‐9 (F) in the laser‐irradiated tissue between wild‐type (WT) and TNFα‐null (KO) mice at each timepoint. Bars, s.e.m.; Statistical analysis was performed by using the one‐way anova

mRNA expression of IL‐6, VEGF‐A, MCP‐1, TGFβ1 exhibited a peak of upregulation on Day 1 or Day 3 and then declined (Figure 3A–D). mRNA expression of these all factors examined was not altered by TNFα gene ablation at each timepoint (Figure 3A–D). Because a neutrophil secretes MMP2 and MMP9, both of which are capable of basement membrane degradation and NV development, we also evaluated mRNA expression of these MMPs. The expression level of both MMP2 and MMP9 was not influenced by lacking TNFα (Figure 3E,F).

### 3.4 Growth factor expression in macrophages and in neutrophils

**FIGURE 4:**
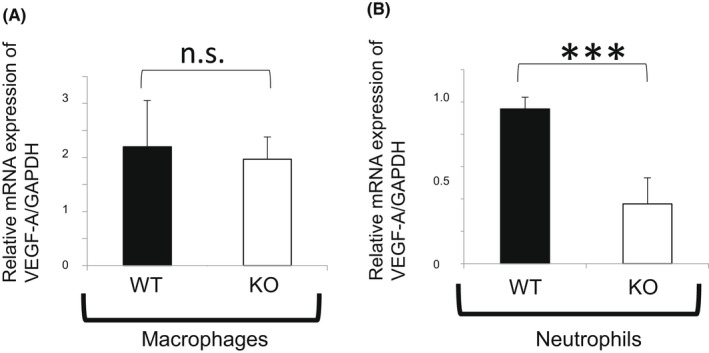
Expression of vascular endothelial cell growth factor‐A (VEGF‐A) in cultured macrophages and neutrophils. Expression of VEGF‐A was un unaffected by the loss of tumour necrosis factor α (TNFα) in cultured macrophages. That was suppressed in a KO neutrophil as compared with each of their WT counterparts. Bars, s.e.m.; n.s; not significant, ***p < 0.001 by the Mann–Whitney *U*‐test

Expression of VEGF‐A was un unaffected by the loss of TNFα in cultured macrophages. That was, in turn, suppressed in a KO neutrophil as compared with each of their WT counterparts (Figure 4).

### 3.5 Apoptosis in CNV tissue and TNFα‐treated HRMEC culture

**FIGURE 5:**
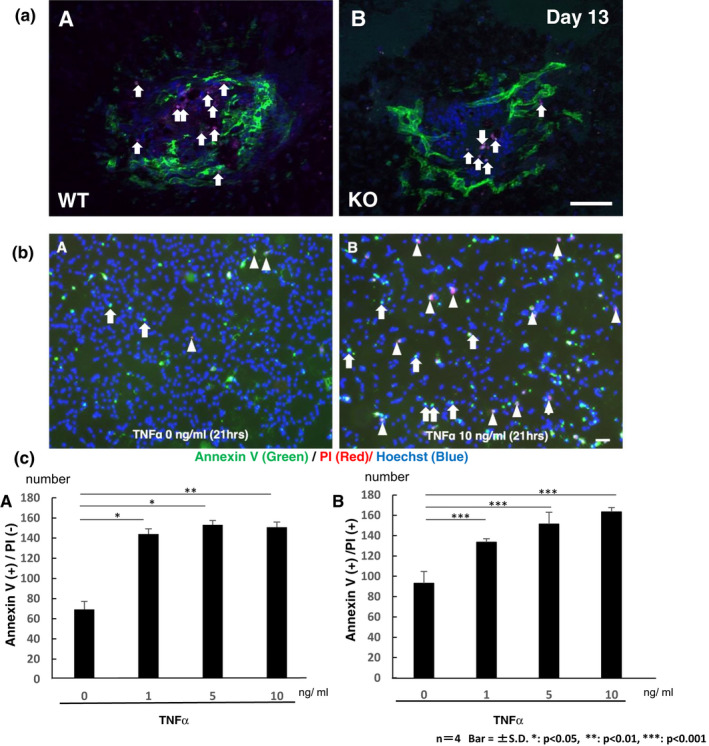
Apoptosis in CNV tissue and tumour necrosis factor α (TNFα)‐treated human retinal microvascular endothelial cells (HRMECs) culture (a). Loss of TNFα enhances the expression of cleaved caspase 3 (apoptosis marker) during the regression of laser‐induced choroidal neovascularization (CNV). Immuno‐detection of PECAM‐1 (vascular endothelium marker, FITC) detects laser‐irradiation induced CNV in a similar fashion on Day 13. While cells with cleaved caspase 3 (Rhodamin) are more frequently in a WT tissue (a) as compared with a KO mouse at this timepoint. Nuclei were labelled with DAPI. Bar, 50 μm (b). Exogenous TNFα induces apoptosis in HRMECs. Immuno‐detection of AnnexinV (Green) and propidium iodide (PI, Red) in HRMECs at 21 h post‐TNFα (10 ng/ml) addition. Nuclei were labelled with Hoechst. Arrows indicate early apoptotic cells with Annexin V‐plus/PI‐minus and arrowheads late apoptotic cells with Annexin V‐plus/PI‐plus. (c). The numbers of Annexin V‐plus/ PI‐minus early apoptotic cells increase in a TNFα‐dose‐dependent manner. Bar, 100 μm c. TNFα addition also increases the numbers of Annexin V‐plus/PI‐plus late apoptotic cells at and over the concentration of 1 ng/ml. Statistical analysis was performed by using the Student's t‐test with a significant level of *p* < 0.05. Bars, s.e.m.; *p < 0.05, **p < 0.01, ***p < 0.001

Immuno‐detection of PECAM‐1 (CD31)‐positive CNV was labelled with FITC and cleaved caspase 3‐expressing apoptotic cells were detected with Rhodamin. Distribution of cleaved caspase 3‐labelled apoptotic cells was more frequently observed in the laser‐irradiated tissue in a WT mouse as compared with a KO mouse (Figure 5a) on Day 13 when immunohistochemically observed in flat‐mounted samples. Population of vascular endothelial cells seemed similar to each genotype of mice at this timepoint.

Because delayed regression of CNV in KO mice could be due to the impaired apoptosis in vascular endothelial cells, we examined if TNFα affects cell death in HRMECs. At 6 h after adding TNFα (1.0, 5.0 and 10 ng/ml) to the medium, the number of annexin V‐positive and PI‐negative cells was similar between TNFα‐plus culture and the control culture (data not shown). At 21 h post‐TNFα supplementation, cell counting indicated that the numbers of annexin V‐positive and PI‐negative (early apoptotic) cells (white arrows) and of annexin V‐positive and PI‐positive (late apoptotic) cells (white arrowheads) were both increased in a concentration‐dependent manner (Figure 5c).

### 3.6 Effects of exogenous TNFα on tube‐like structure of HRMECs in vitro

**FIGURE 6:**
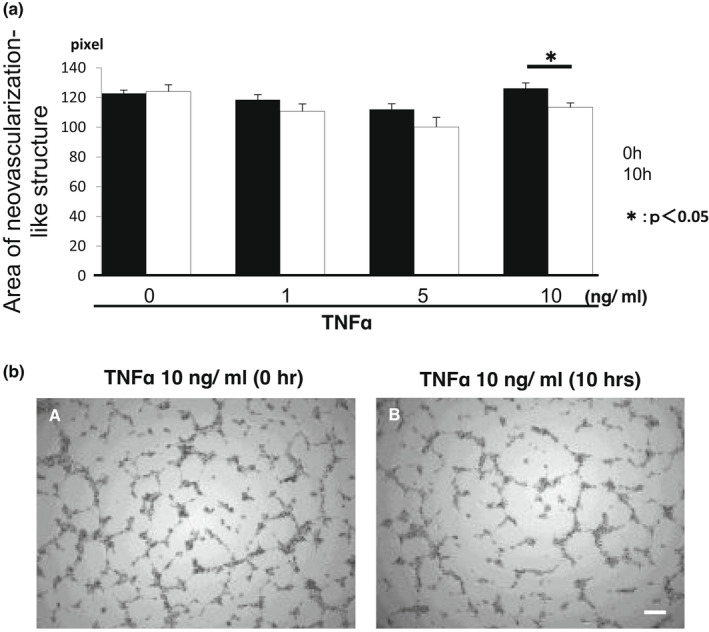
Effect of exogenous tumour necrosis factor α (TNFα) on the vessel‐like structure developed by human retinal microvascular endothelial cells (HRMECs). Cultrex RGF BME (10 μl) was placed in wells of a 96‐well plate and allowed to set for 30 min in a CO2 incubator. HRMEC cells (2 × 10^4^ cells in 70 μl/well) were seeded and incubated for 10 h for induction of tube‐like structure. The cultures were incubated for 10 h in the presence or absence of TNFα (1, 5, 10 ng/ml). (a). TNFα dose‐dependently inhibits HRMECs tube formation. Bars, s.e.m.; *p < 0.05. by the Student's *t*‐test. (b). The representative images show cells at 0 or 10 h post‐TNFα addition. Bar, 100 μm

The effect of TNFα addition on the tube‐like structure of HRMECs was observed. We previously reported human umbilical vein endothelial cells on the fibroblast feeder layer did not form the tube‐like structure in the TNFα‐containing medium.^20^ Here, we administered TNFα after allowing the cells to form vessel‐like structure in order to know the effect of TNFα on the tube‐like structure already formed without the ligand. After the cells formed tube‐like structure, TNFα at the final concentrations of 1.0, 5.0 or 10 ng/ml was administered and the morphology of the tube‐like structure was observed until 10 h under time‐lapse microscopy. The tube‐like structure of the cells was degraded presumably by cell death, as observed under time‐lapse microscopy. Free National Institutes of Health software Image‐J Computer software‐assisted procedure was applied to measure the total length of the tube‐like structure in the center area of each well. Adding TNFα at the concentration of 10 ng/ml reduced the total area of tube‐like structure with a statistical significance at 10 h (Figure 6a,b).

## 4 DISCUSSION

The size of the CNV was significantly larger in a KO mouse as compared with a WT mouse at week 3, that is, during the regression period after the peak of the size of the CNV. The finding suggests either that the loss of TNFα either promotes the growth of new vessels or that it inhibits the regression of CNV. Although it is to be noted that the loss of an inflammatory growth factor does not suppress the growth of CNV, our original hypothesis was TNFα deletion might promote its growth.

We previously reported that TGFβ/Smad‐dependent infiltration of macrophages is critical to the development of laser‐induced CNV in mice.^8^ Thus, we focused on the effects of the loss of TNFα on the level of inflammatory cell infiltration and the role of inflammation‐related growth factors/cytokines in the laser‐irradiated tissue with the prolonged presence of CNV. We showed here an increased population of neutrophils in the tissue in the absence of TNFα on Days 1, 3 and 5. However, neutrophils almost disappeared in the laser‐irradiated tissue in our model of a WT mouse, as previously reported.^27^ In an injured tissue, infiltration of neutrophils in the early phase of the tissue response to injury is followed by the invasion of macrophages, both of which are capable of secretion of angiogenic factors.^28^ Neutrophils and macrophages contribute to the development of neovascular tissue in various settings.^29^, ^30^, ^31^, ^32^, ^33^, ^34^, ^35^ It was reported that neutrophil depletion induced by an anti‐murine neutrophil‐antibody injection reduced CNV formation in mice.^36^ Thus, the promotion of growth of CNV by the loss of TNFα could be attributable to neutrophil infiltration. However, the mechanism of more marked infiltration of neutrophils in the KO tissue is to be clarified. Inflammatory cells are considered to disappear from the local tissue, presumably by the mechanism of apoptosis. TNFα, as a pro‐inflammatory cytokine, exerts its multiple biological activities by signalling via its two receptors, TNFR‐1 and TNFR‐2.^37^ Gene knockout of TNFR‐2 reportedly suppressed the growth of laser‐induced CNV in association with less macrophage infiltration to the local tissue in mice, while TNFR‐1‐null mice exhibited a larger CNV as compared with a WT mouse.^19^ Murray et al. reported that TNFα induces apoptosis in neutrophils mainly via TRFR‐1.^38^ This is consistent with a report that only TNF‐R1 contains a cytoplasmic death domain and may directly induce apoptosis.^39^ Ugan et al. reported that anti‐TNFα antibody administration blocks neutrophil apoptosis in patients with ankylosing spondylitis.^40^ Based on the findings in these reports by investigators, we consider that the increased population of neutrophils in the local laser‐irradiated tissue might be due to the impairment of cell clearance by apoptosis.

In the present study, we evaluated mRNA expression level of angiogenic factors, for example, IL‐6 and VEGF‐A and TGFb1, and a major factor involved in macrophage infiltration, MCP‐1. MMP2/9 are both involved in neovascularization development and growth.^41^, ^42^ Neutrophil reportedly expresses MMP9 that is a promoting factor for neovascularization.^43^ The expression of MMP2 and MMP9 mRNA was unchanged by the loss of TNFα. In the present situation, augmented infiltration of neutrophils did not correlate to the alteration of the expression level of pro‐angiogenic components, that is, VEGF‐A, TGFβ1 MMP‐2 and MMP9, in the irradiated tissue in KO mice. Current in vitro experiments showed that expression of VEGF‐A in neutrophils was suppressed by the loss of TNFα in vitro, while that in vivo treated tissue was unchanged in a KO animal.

TNFα is reportedly accelerating apoptosis of vascular endothelial cells or inhibits cell senescence.^44^, ^45^, ^46^, ^47^ We next hypothesized that the larger CNV in KO mice was attributable to an impaired regression of new vessels due to less apoptosis in vascular endothelial cells. To explore this hypothesis, we performed immuno‐detection of cleaved caspase 3, a critical component in the process of TNFα‐induced apoptotic cell death in the laser‐irradiated tissue. More cells with immunoreactivity for cleaved caspase 3 were seen among CD31‐labelled choroidal neovascularization in a KO irradiated tissue as compared with a WT mouse on Day 13. We then examined the effect of exogenous TNFα on the level of apoptosis of cultured HRMECs. We previously reported that adding exogenous TNFα blocked the formation of CD31‐labelled tube‐like structure by human umbilical vein endothelial cells (HUVECS) cultured on fibroblast feeder layer.^20^ However, unfortunately, we failed to examine the effects of exogenous TNFα on the apoptotic cell death of HUVECs. In the present study, we showed that exogenous TNFα induced apoptosis in cultured HRMECs and also showed that it promoted the disappearance of tube‐like structure of HRMECs cultured on matrigel. These in vitro findings suggest that the mechanism of inhibition of regression of laser‐induced CNV in the absence of TNFα might include suppression of vascular cell apoptosis in the loss of cell death signal by TNFα.

In conclusion, we showed here that the loss of TNFα does not suppress CNV development after laser irradiation and prolonged the survival of the laser‐induced CNV in association with reduced vascular cell apoptosis and accumulation of neutrophils in the local tissue. The findings suggest that delayed regression of CNV in KO mice might be attributable to an impairment of apoptotic cell death of vascular endothelium in KO mice. Antibody neutralization of TNFα is beneficial to treat macular neovascularization, and, thus, total gene knockout does not represent partial inactivation of the ligand. The regression of CNV might be followed by the accumulation of scar‐like fibrotic tissue that causes visual function in the later interval after treatment.^48^ We, therefore, examined the degree of the formation of fibrovascular tissue in the area of CNV in a 35 days sample by using immune‐detection of αSMA and collagen type I, the two major fibrosis markers, and did not observe the difference of the protein expression pattern of these components between WT and KO mice. We previously reported that TNFα‐null eye cornea showed more severe inflammatory fibrosis during healing after an alkali exposure,^24^ which differs from the current CNV model. The reason for discrepancy is to be investigated, but the severity of tissue damage in these two injury models could affect the degree of tissue fibrosis in the later phase of healing.

## AUTHOR CONTRIBUTIONS

**Hiroki Iwanishi:** Data curation (lead); formal analysis (lead); funding acquisition (lead); investigation (lead); methodology (lead); project administration (equal); resources (lead); software (lead); visualization (lead); writing – original draft (lead). **Osamu Yamanaka:** Conceptualization (supporting); methodology (supporting). **Takayoshi Sumioka:** Conceptualization (supporting); investigation (supporting); methodology (supporting); writing – original draft (supporting). **Masayasu Mijajima:** Resources (supporting). **Shizuya Saika:** Conceptualization (lead); formal analysis (lead); investigation (supporting); methodology (lead); project administration (lead); software (supporting); supervision (lead); writing – original draft (equal); writing – review and editing (equal). **Shingo Yasuda:** Data curation (equal).

## CONFLIC OF INTERESTS

None for all authors.

## FUNDING INFORMATION

This study was supported by the competitive research grant from Bayer Japan (Grant #; BASJ20160403005).

## Data Availability

Data available on request from the authors.
